# Immunomodulation of human T cells by microbubble-mediated focused ultrasound

**DOI:** 10.3389/fimmu.2024.1486744

**Published:** 2024-10-22

**Authors:** Ana Baez, Davindra Singh, Stephanie He, Mehri Hajiaghayi, Fatemeh Gholizadeh, Peter J. Darlington, Brandon Helfield

**Affiliations:** ^1^ Department of Biology, Concordia University, Montreal, QC, Canada; ^2^ Department of Physics, Concordia University, Montreal, QC, Canada

**Keywords:** cancer immunotherapy, human peripheral blood mononuclear cells, membrane permeabilization, cytokine release, cavitation

## Abstract

While met with initial and ground-breaking success targeting blood borne cancers, cellular immunotherapy remains significantly hindered in the context of solid tumors by the tumor microenvironment. Focused ultrasound, in conjunction with microbubbles, has found tremendous potential as a targeted and local drug/gene delivery technique for cancer therapy. The specific immunomodulating effects of this technique on immune cells, including T-cells, remain unexplored. Here, with freshly isolated human immune cells, we examine how focused ultrasound can viably modulate immune cell membrane permeability and influence the secretion of over 90 cytokines, chemokines and other analytes relevant to a potent immune response against cancer. We determine that microbubble-mediated focused ultrasound modulates the immune cell secretome in a time-dependent manner – ranging in ~0.1-3.6-fold changes in the concentration of a given cytokine compared to sham controls over 48 hours post-treatment (*e.g.* IL-1β, TNF-α, CX3CL1, CCL21). Further, we determine the general trend of a negative correlation between secreted cytokine concentration and viable ultrasound-assisted membrane permeability with negligible loss of cell viability. Taken together, the data presented here highlights the potential of microbubble-mediated focused ultrasound to viably enhance T-cell permeability and modulate key pro-immune pathways, offering a novel approach to augment targeted cellular therapies for solid tumors.

## 1 Introduction

Cancer immunotherapy involves leveraging the immune system to target and eliminate cancer cells, and this technique has shown exciting and ground-breaking results with strategies ranging from immune checkpoint inhibitors, adoptive cell transfer, and therapeutic vaccines (1, 2). In particular, cellular immunotherapies - in which immune cells (T, NK) are isolated, expanded and re-introduced into patients – have met initial success in treating blood-borne cancers yet face challenges in solid tumors due to the suppressive nature of the tumor microenvironment (TME) (3, 4). Indeed, in the native tumor setting both endogenous and exogenously introduced T cells typically fail to irradicate cancer cells in such solid tumors even in situations with sufficient T cell infiltration and antigen-recognition (*i.e.* an immunologically ‘hot’ tumor). The TME is a complex network composed of various cellular and molecular components that create a hostile environment for immune cells and thus antitumor T cells progressively acquire a dysfunctional state during tumor progression, ultimately enabling tumor immune-evasion (5, 6). Factors such as immunosuppressive cells (*e.g.* regulatory T cells, tumor-associated macrophages, and myeloid-derived suppressor cells) along with inhibitory cytokines contribute to this immunosuppressive milieu, impairing both natural and therapeutic immune responses (7). Strategies to overcome T-cell anergy and restore their antitumor functionality within the TME is an ongoing and active area of research, and are essential for enhancing the efficacy of cancer immunotherapies (8, 9).

Focused ultrasound technology in combination with microbubbles presents a promising approach to enhance antitumor response and intrinsically modulate the TME. Microbubbles are clinically available intravascular contrast agents, typically employed in echocardiography to enhance vasculature contrast (10). These microbubbles are small (1-8 µm) gas-filled and encapsulated bubbles that vibrate within an ultrasound field, expanding and contracting about their equilibrium size (11). Under specific acoustic conditions, vibrating microbubbles can be made to exert local bioeffects on neighboring vasculature and tissue; including the transient modulation of endothelial membrane permeability (12) and calcium influx [both membrane perforation-induced and channel-mediated (12–15)], as well as stimulation of inflammatory signaling (16–18). One key advantage is their ability to temporarily and reversibly open vascular barriers like the blood-brain barrier (19), enabling localized immunomodulating drug delivery and minimizing systemic side effects (20, 21). This combination of improved drug delivery and immune modulation positions ultrasound and microbubble therapy as a multifaceted approach to overcoming immunosuppressive barriers within the TME.

Despite these potential benefits, the detailed mechanisms by which microbubbles interact with immune cells, particularly T-cells, remain poorly understood. Given the central role of T-cells in mediating immune responses against cancer, it is crucial to elucidate these interactions to optimize ultrasound-assisted immunotherapy outcomes (22).

Here, we aim to explore how microbubble-assisted focused ultrasound affects T cells, both in terms of a targeted T cell drug delivery technique as well as a more general immunomodulation tool. First, we assess the ability of focused ultrasound to viability increase T-cell membrane permeability to an otherwise impermeable macromolecule using two human T-cell models (immortalized and freshly isolated tissue). Next, we examine how this treatment affects the immune cell secretome over time up to 48 hours post-treatment, including the release of T-cell activating factors and immune cell chemotactic signals. Finally, we assess the relationships between membrane permeability enhancement and T-cell cytokine release signaling and place our results within the context of using microbubble-mediated focused ultrasound as a cancer immunotherapeutic tool.

## 2 Materials and methods

### 2.1 Isolation of human peripheral blood mononuclear cells

After obtaining informed, signed consent from healthy participants, blood was collected and approved by the Concordia University Human Research Ethics Committee, following the Declaration of Helsinki guidelines (certificate 30009292). Participants’ health status was verified through self-reporting during a semi-structured interview. Individuals under 18, those with certain medical conditions, or those taking specific medications were excluded. Blood draws were postponed if the participant had used recreational drugs or received a vaccination within the last two weeks.

For the PBMC isolation, heparinized peripheral blood was diluted with 1X PBS in a 1:1 ratio. In 50-ml conical tubes, 30 ml of the diluted blood was carefully layered over 15 ml of lymphocyte separation solution (Wisent Bioproducts, CA). The samples were centrifuged at 624g for 30 minutes at room temperature. Mononuclear cells were then collected into another 50-ml tube and centrifuged at 433g for 15 minutes with 25 ml of PBS. After discarding the supernatant, the pellet was washed with 25 ml of PBS and centrifuged again at 400g for 12 minutes. The PBMCs were cultured in a medium comprising 10% heat-inactivated fetal bovine serum (FBS; Wisent Inc., Montreal, QC, Canada) in Roswell Park Memorial Institute (RPMI 1640) medium, supplemented with 1mM penicillin, streptomycin, and 2mM GlutaPlus (Wisent Inc., QC, Canada) at 37° cell culturing.

### 2.2 Cell culturing

The Jurkat E6 human cell line was obtained from the American Type Culture Collection (ATCC #TIB-152). Both Jurkats and PBMCs were maintained in RPMI medium supplemented with 10% fetal bovine serum (FBS) and grown in 75 cm^2^ tissue culture flasks (Corning, #430641U) at 37°C, 5% CO2 in a humidified incubator (Thermo Fisher, CA, USA). Jurkat T-cells were passaged every three days or when cells reached approximately 90% confluency, and cell passages of 6-15 were used for all experiments. The number of viable cells was quantified using a trypan blue measurement on the cell counter Countess 3 (Thermofisher, CA, USA). Cells were then activated for a subset of the experiments using 1 µg/ml anti-CD3 and 3 µg/ml of anti-CD28 (Cytek Biosciences, CA, USA) for Jurkat cells and Immunocult™ (Stem cell technologies, CA, USA) for PBMCs.

### 2.3 Ultrasound treatment and experimental procedure

**Figure 1:**
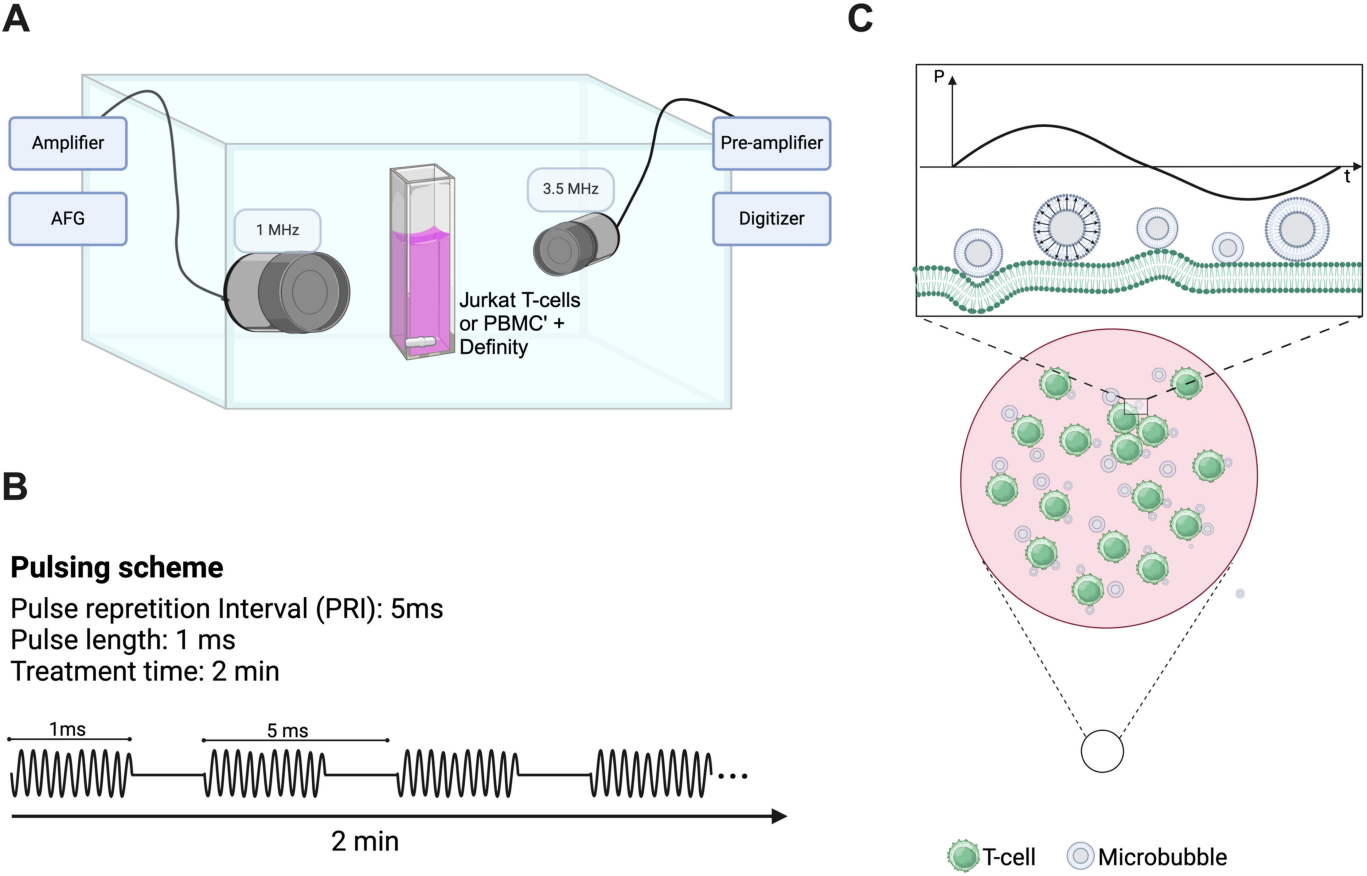
Custom-designed ultrasound treatment setup and protocol. **(A)** A custom-made water tank was maintained at 37°C and consisted of a cell suspension chamber position at the co-focus of two co-aligned transducers (1 MHz therapy transducer and 3.5 MHz passive cavitation detector). Suspensions of clinical Definity™ microbubbles were incubated with either human Jurkat T-cells or freshly isolated human peripheral blood mononuclear cells within the suspension chamber in either the presence or absence of FITC-dextran (10 kDa). This chamber was placed atop of magnetic stir plate to ensure homogeneity of solution, and sonicated for 2 minutes with a 1 MHz, 1000 cycle, 20% duty cycle sequence ranging in acoustic peak-negative pressure from 208-563 kPa [schematic shown in panel **(B)**]. **(C)** Schematic representation of vibrating microbubbles adjacent to immune cells (*e.g.* T-cell) within the suspension chamber.

We designed a custom-built acoustic tank to examine the effects of ultrasound on populations of immune cells (23) (see **Figure 1**). Briefly, an acrylic tank filled with gas-equilibrated distilled water was kept at a constant temperature of 37°C using a VWR immersion heater circulator (model 1120, Radnor, PA, USA). The tank setup included a sample chamber crafted from acrylic with mylar windows (25 µm thick) for ultrasound wave transmission and two aligned single-element transducers. The single-element therapy transducer (Olympus) operated at 1 MHz with a focal length of 25.4 mm and F-number of 1.33, delivering 1000 cycles of ultrasound with a pulse repetition interval of 5 ms, resulting in a duty cycle of 20%. The peak-negative pressures examined here range from 208-563kPa, as calibrated in free space within a separate water tank (HGL-0200, ONDA, Sunnyvale, CA, USA). The passive single-element transducer (Olympus; 3.5 MHz center frequency, unfocused) was used to record microbubble scattering to assess the presence of stable and inertial cavitation. These cavitation categories have been correlated to physical bubble behavior, ranging from stable vibration to bubble disruption. Echoes were amplified (AU-1579, 0.7–200 MHz, MITEQ, Hauppauge, NY, USA), bandpass-filtered, and then digitized (Gage Razor Express CompuScope, Lockport, IL, USA) for off-line analysis using custom MATLAB software (Mathworks, Natick, MA, USA). A Hamming window was applied to the RF data before obtaining fast Fourier transforms to assess frequency response. Joint time-frequency analysis was performed over a window size of 50 ms and 90% overlap. Broadband emissions, quantified here as the integrated signal arising at non-harmonic bands near the center frequency of the receive transducer, were used as an estimate for inertial cavitation, as has been done previously (24, 25). The inertial cavitation dose is defined here as the cumulative integrated power over the course of the 2 minute treatment.

The samples were prepared with either naïve or activated cells (either Jurkat or PBMCs) at a final concentration of 5.8 x 10^6^ cells/ml in a total volume of 3 ml, with the addition of 38 µl of Definity™ microbubbles (cell-to-bubble ratio of 1:217) in complete medium. Indeed, inactivated T-cells possess a fundamentally different cellular phenotype and surface expression levels than those that are activated, a potential factor in its response to ultrasound. Definity™ microbubbles are clinically and commercially available, and robustly characterized to consist of a concentration of ~10^10^ bubbles/ml and a volume-weighted distribution peaking at ~2 and ~7 µm in diameter (26). All experiments consisted of freshly activated Definity™ at room temperature to ensure consistent microbubble populations (27). We performed two main experiments. A subset of experiments consisted of sample cocktails containing 5 mg/ml 10 kDa FITC-dextran (which acts here as a surrogate drug) in which permeability to an otherwise non-permeable macromolecule is the main readout. The second set of experiments was conducted in the absence of any fluorescent molecule to assess cytokine/chemokine release. A magnetic stir bar, placed within the sample chamber, facilitated constant stirring of the sample to ensure a homogeneous distribution of both microbubbles and cells. Following a 30-second wait for system equilibration, we proceeded with the ultrasound treatment for 2 minutes. For all experiments, ultrasound sham treatments were conducted, in which all other factors and cell manipulations remained the same. It is worth noting here that the acoustic peak-negative-pressure was varied as it is one of the main factors that influences the extent of microbubble vibration and whether or not it will undergo inertial collapse (11), and preliminary tests were conducted to ensure the selected pulse repetition interval and number of cycles were not destructive.

### 2.4 Flow cytometry analysis and staining

After treatment using FITC-dextran, cells were washed three times and resuspended in 1X PBS. PBMCs were further stained with anti-CD3-Cy7 and anti-CD4-BV421 (Thermofisher Scientific, CA, USA) as per manufacturer instructions to assess T-cell populations. For both Jurkat T cells and PBMCs, propidium iodide (PI; ThermoFisher) was added 1 minute before analysis in the FACS Melody flow cytometer (BD, USA, CA) to assess cell viability. Sham treatments, as well as single and double fluorescent molecule-absent samples, were used to delineate the 2-channel gating. The cell populations that exhibited both FITC+ and PI- signals were considered viably permeated via ultrasound and microbubbles. These cells (FITC+ and PI-) were then further subdivided to quantify T-cells specifically via CD3+CD4- and CD3+CD4+ cells obtained by double and triple-stained cells (FITC+ and Cy7 as well as FITC+, Cy7 and BV421 respectively). Here, the CD3+CD4- population was considered as a surrogate for the CD8+ population.

### 2.5 Human 96-plex cytokine/chemokine profiling

Supernatants from PBMCs were collected at different time points post-treatment with 208kPa-563kPa (3h, 6h, 12h, 24h and 48h). Chemokine/cytokine profiling was performed by Eve Technologies (Alberta, Canada) using a multiplex platform (Luminex™ 200 system; Luminex, Austin, TX, USA), which relies on capture-antibody conjugated fluorescent beads passing through a two-layer system. Specifically, we assayed the Human Cytokine/Chemokine 96-Plex Discovery Assay panel from MilliporeSigma (Burlington, Massachusetts, USA), which contains the following biomarkers: 6Ckine | APRIL | BAFF | BCA-1 | CCL28 | sCD137 | sCD40L | CTACK | CXCL16 | EGF | ENA-78 | Eotaxin | Eotaxin-2 | Eotaxin-3 | sFas | sFasL | FGF-2 | FLT-3L | Fractalkine | GCP-2 | G-CSF | GM-CSF | Granzyme A | Granzyme B | GROα | HMGB1 | I-309 | IFN-α2 | IFNβ | IFN*ω* | IFNγ | IL-1α | IL-1β | IL-1RA | IL-2 | IL-3 | IL-4 | IL-5 | IL-6 | IL-7 | IL-8 | IL-9 | IL-10 | IL-11 | IL-12p40 | IL-12p70 | IL-13 | IL-15 | IL-16 | IL-17A | IL-17E/IL-25 | IL-17F | IL-18 | IL-20 | IL-21 | IL-22 | IL-23 | IL-24 | IL-27 | IL-28A | IL-29 | IL-31 | IL-33 | IL-34 | IL-35 | IP-10 | I-TAC | LIF | Lymphotactin | MCP-1 | MCP-2 | MCP-3 | MCP-4 | M-CSF | MDC | MIG/CXCL9 | MIP-1α | MIP-1β | MIP-1δ | MIP-3α | MIP-3β | MPIF-1 | PDGF-AA | PDGF-AB/BB | Perforin | RANTES | SCF | SDF-1 | TARC | TGFα | TNFα | TNFβ | TPO | TRAIL | TSLP | VEGF-A. Sample preparation was done as per manufacturers’ instruction including quality controls to ensure the validity of the results obtained. Assay sensitivities of these markers range from 0.05 – 100 pg/ml for the 96-plex. Data was processed such that fold changes of the different cytokines were calculated relative to sham ultrasound treatments at each time point, and only those that displayed statistical significance at one or more time-points is presented here. Gene enrichment via over-representation analysis was performed to ascertain the extent to which focused ultrasound modulation of immune cells aligns with known biological signaling pathways. Significant enrichment was quantified via a false discovery rate (FDR), and only those with an FDR less than 0.05, based on p-values after multiple comparison corrections, are shown. A subset of this cytokine dataset was validated using individual ELISA kits (Thermofisher), as per manufacturer’s instructions (see **Supplementary Figure S1**).

### 2.6 Statistical analysis

All experiments described were performed in 2–4 biological replicates. Statistical analyses were performed using Prism 9 (GraphPad Software). Statistical significance was determined using two-tailed, Mann-Whitney test or One-way ANOVA for multiple comparisons. Simple linear regression was performed to correlate cytokine fold change and cell permeability. Results are expressed as mean ± SEM, and *p* < 0.05 was considered statistically significant.

### 2.7 Protein interaction map and KEGG pathway analysis

A protein interaction network map was generated using the STRING database version 12, which integrates known and predicted protein-protein interactions from various sources, including curated databases, experimental data, and text mining. Interactions were visualized with confidence scores, and different colors were used to represent the type of evidence supporting the interactions (blue for curated databases, purple for experimental data, and green for text mining).

Additionally, we used the KEGG (Kyoto Encyclopedia of Genes and Genomes) pathway database to identify and categorize the pathways in which the cytokines are involved. This allowed us to map the cytokines to key signaling pathways, such as TNF, NF-κB, and T cell receptor signaling providing a clearer understanding of their biological roles and potential impact on immune modulation.

## 3 Results and discussion

**Figure 2:**
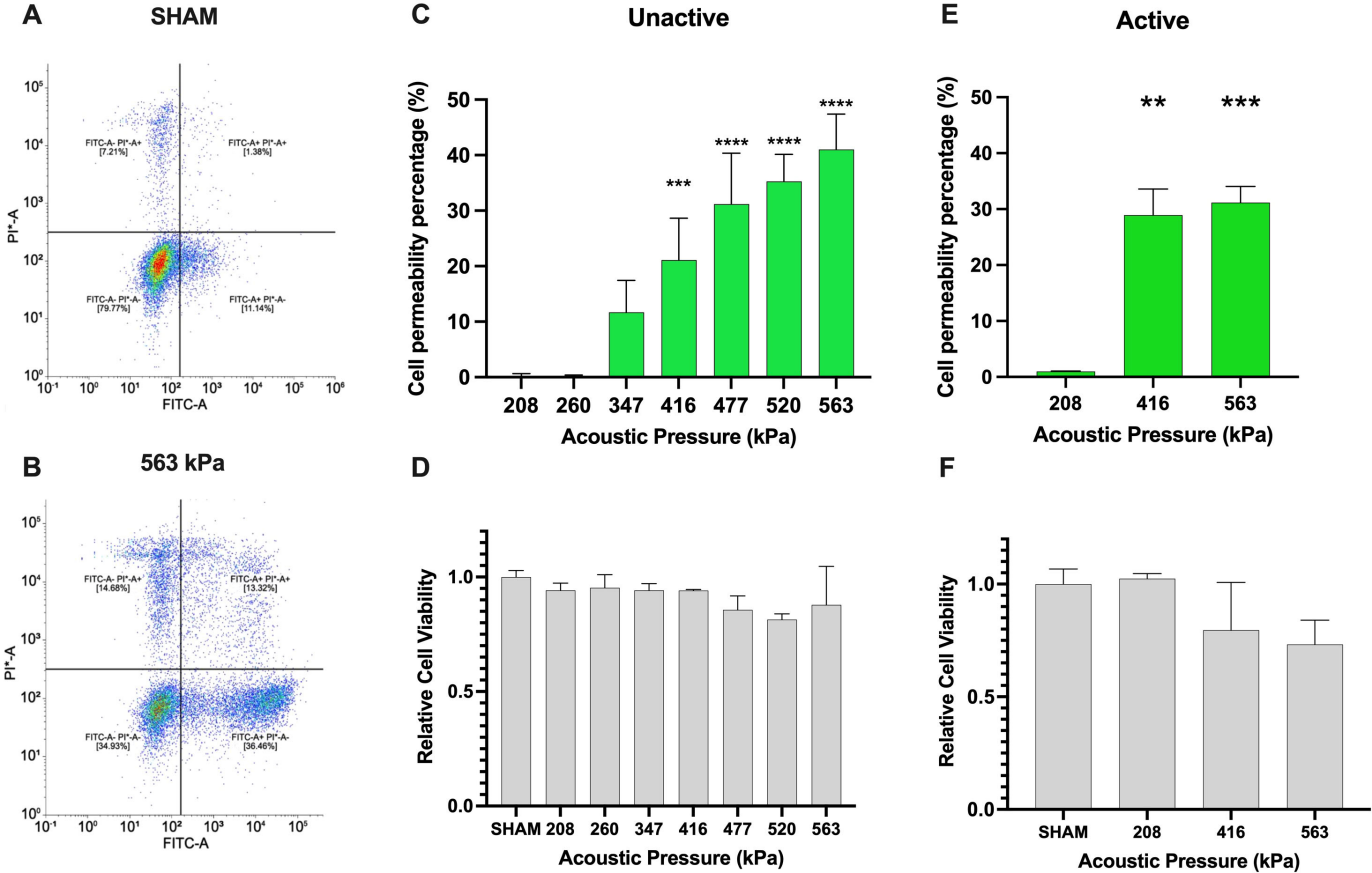
Ultrasound-assisted viable macromolecule delivery to Jurkat T-cells is a function of acoustic pressure. To highlight our gating strategy, we display the flow cytometry gating after **(A)** SHAM (no ultrasound) and **(B)** 563kPa treatment. **(C)** Ultrasound-assisted delivery of an otherwise impermeable macromolecule (10 kDa) to unactivated Jurkat T-cells displays up to 40% efficiency (*p*<0.001), increasing with acoustic transmit pressure and **(D)** accompanied by a small loss of cell viability from 86-100% depending on the condition. Panels **(E, F)** similarly demonstrate ultrasound-assisted delivery of the macromolecule to anti-CD3 anti-CD28 activated Jurkat T-cells over a subset of acoustic pressure, highlighting the similar trend of up to 32% percent delivery efficiency with viability ranging from 75-100% (*p*<0.001). Data is represented as mean ± standard error of the mean at least *n*=3 independent samples. ** p<0.01, *** p<0.001, **** p<0.0001.

First, we examine the extent to which ultrasound and clinical agent Definity™ can enhance the cell membrane permeability while preserving the viability of Jurkat T-cells – illustrative examples of which are shown in **Figures 2A, B**. Here, successful membrane permeation is quantified via uptake of the 10 kDa FITC-labeled dextran (FITC+ half-space), and cell viability is assessed through PI dye exclusion (PI- half-space). The overall quantitative analysis of both sham-corrected viable and permeated cells (FITC+ ∩ PI-) is shown in **Figure 2C** for unactivated Jurkat T-cells and **Figure 2E** for activated ones as a function of acoustic pressure, along with the overall cell viability (PI-) in **Figures 2D, F**, respectively. For the unactivated Jurkats, the ultrasound-stimulated permeability significantly increased from negligible amounts at 208 kPa up to 41.0% at 563 kPa (*p* < 0.0001), while maintaining high levels of viability across all acoustic pressures similar to the sham control, albeit demonstrating a slight decrease at the highest acoustic pressure employed here (83.8% viability). Despite the fact that absolute-value comparison among other population-based studies and cell lines is challenging due to differences in acoustics and microbubble physics, the increasing efficacy and decreasing viability with harsher ultrasound conditions is broadly consistent with other works (23, 28–30), including the very few that have previously examined Jurkat T-cells (31, 32). Indeed, these limited previous works examined naïve unactivated Jurkats, characterized by a spherical shape and a large nucleus to cytosol ratio. Given the potential for plasma membrane biophysics in influencing ultrasound-assisted membrane permeability, we next repeated these experiments over a subset of acoustic conditions on activated Jurkat cells – as quantified by large IL-2 secretion. First, we confirmed physiologically relevant activation of Jurkat T-cells via IL-2 secretion via ELISA, in which our protocol yielded 40pg/ml of IL-2 compared with undetectable amounts of IL-2 in unactive cells (results not shown). Similar to our unactivated Jurkat results, the cell permeability of activated Jurkat cells increased from 0.94% at 208 kPa to 31.1% at 563 kPa (*p* < 0.001), while maintaining a similar level of viability up to 416 kPa and decreasing at the largest amplitude employed (83.5% viability), as shown in **Figure 2F**.

**Figure 3:**
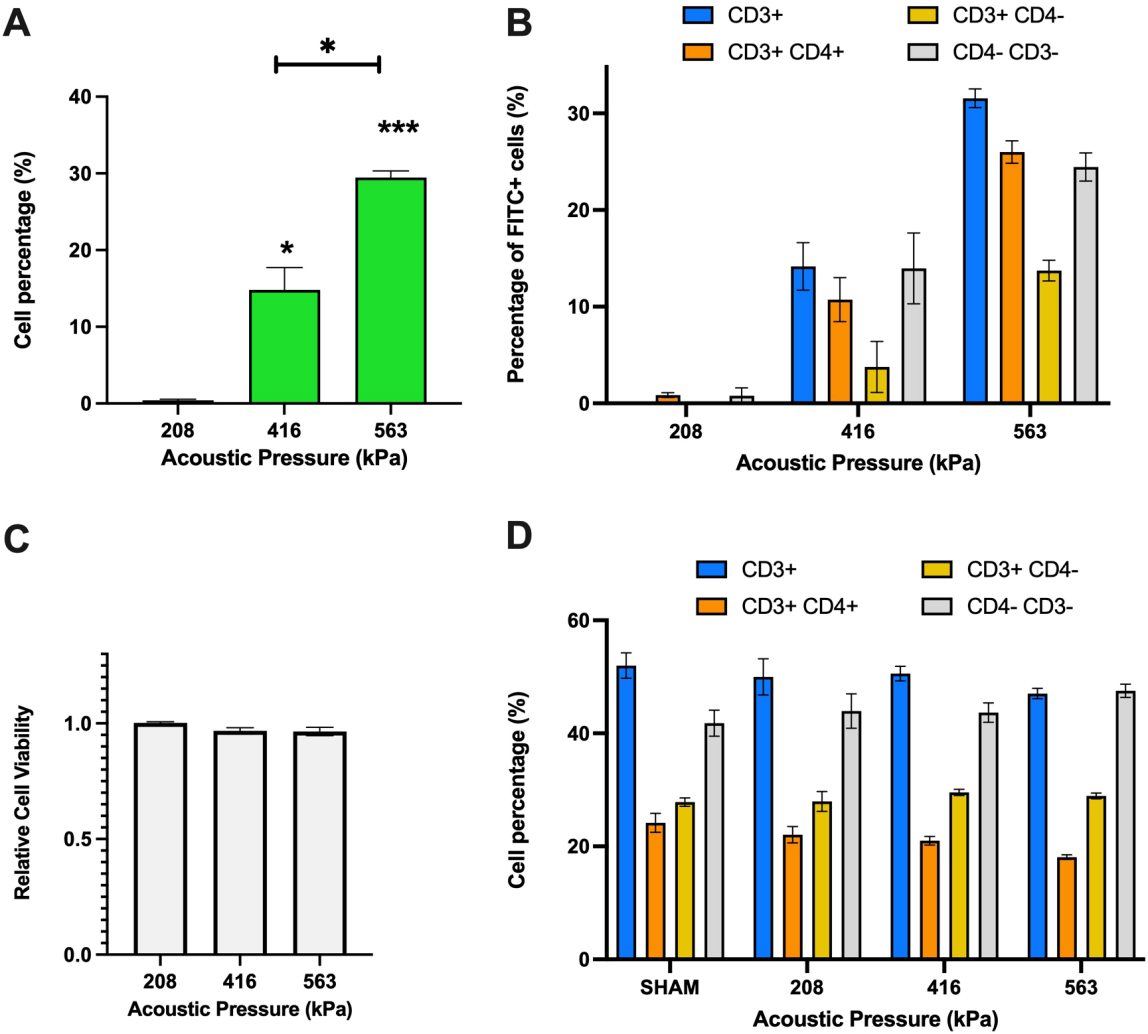
Ultrasound-assisted permeabilization of freshly isolated immune cells reaches similar levels of efficiency as Jurkat T-cells at higher levels of cell viability. **(A)** Ultrasound-assisted delivery of 10 kDa FITC-Dextran to activated PBMCs demonstrates up to 30% efficiency over the acoustic peak-negative pressure range considered here (*p*<0.001). **(B)** The ultrasound-assisted delivery sub-divided into immune cell subset populations highlight similar levels of plasma permeability in CD3+ cells as the overall dataset. **(C)** The cell viability data reveals almost no significant cell viability loss over these conditions. **(D)** The T-cell population and distribution after treatment confirm the homogeneity of individual samples of PMBCs. Data is represented as mean ± standard error of the mean at least n=3 independent samples. * p<0.05, *** p<0.001.

Upon confirming of Jurkat T-cell permeabilization, we next sought to assess microbubble-mediated permeabilization on freshly isolated PBMCs. First, we globally assessed the percentage of cells that were viably permeated by ultrasound, shown in **Figure 3A**. As can be seen in this panel, viable PBMC permeability increased from ~0.4%-29.4% of the total cells over the acoustic conditions employed here (p<0.01 between 563 and 416 kPa groups). Given that PBMCs consist of multiple immune cell types, we next explored, after-the-fact, the uptake of the 10 kDa macromolecule within CD4+ and CD8+ T-cells by staining with Cy7 anti-CD3+ and BV421 anti-CD4+. A limitation of this study is that the presence of CD8 was never measured, and it is assumed here that these cells are represented by the CD3+CD4- population, as mature, peripheral T cells are rarely double negative in healthy volunteers (33, 34). Indeed, **Figure 3B** indicates that at the lowest pressure (208 kPa), the percentage of FITC+ cells were minimal across all T-cell subsets (CD3+, CD3+ CD4+, CD3+ CD4-, and CD4- CD3-). As the pressure increased to 416 kPa, there was a significant rise in viable FITC+ cells, particularly in CD3+ and CD4-CD3- subsets (14.2% and 14.0%). At the highest pressure of 563 kPa, the CD3+ subset exhibited the highest viable uptake of the macromolecule (31.6%), followed by CD3+ CD4+, CD4- CD3-, and CD3+ CD4- subsets (26.0%, 24.5% and 13.7% respectively). These results highlight that increasing acoustic pressure enhances macromolecule uptake in different T-cell subsets. Under this set of acoustic conditions, the PBMC cell viability remained statistically unaffected across the tested acoustic pressures, maintaining high viability levels for all conditions ranging from 96-100% (**Figure 3C**). Indeed, this is further confirmed via **Figure 3D**, which illustrates the distribution of different immune cell subpopulations (CD3+, CD3+CD4+, CD3+CD4−, CD4−CD3−) as measured after ultrasound treatment as a function of transmit acoustic pressure. The proportions of these subpopulations remained relatively consistent, indicating that the acoustic pressures did not negatively selectively affect any specific immune cell type.

It is important to note that the PBMC dataset reported here is broadly consistent with one of the only similar studies, which presented an approximate ~20% uptake of a relatively small macromolecule (calcein; 0.66 kDa) within freshly isolated, but unactivated lymphocytes (35). Indeed, our viability data here (**Figure 3C**) suggests that freshly isolated and activated human immune cells are more robust to ultrasound-assisted permeabilization than either immortalized human T cells (*e.g.* Jurkat as presented in **Figure 2F**) or unactivated ones (35), while exhibiting a similar or elevated level of large macromolecule uptake.

**Figure 4:**
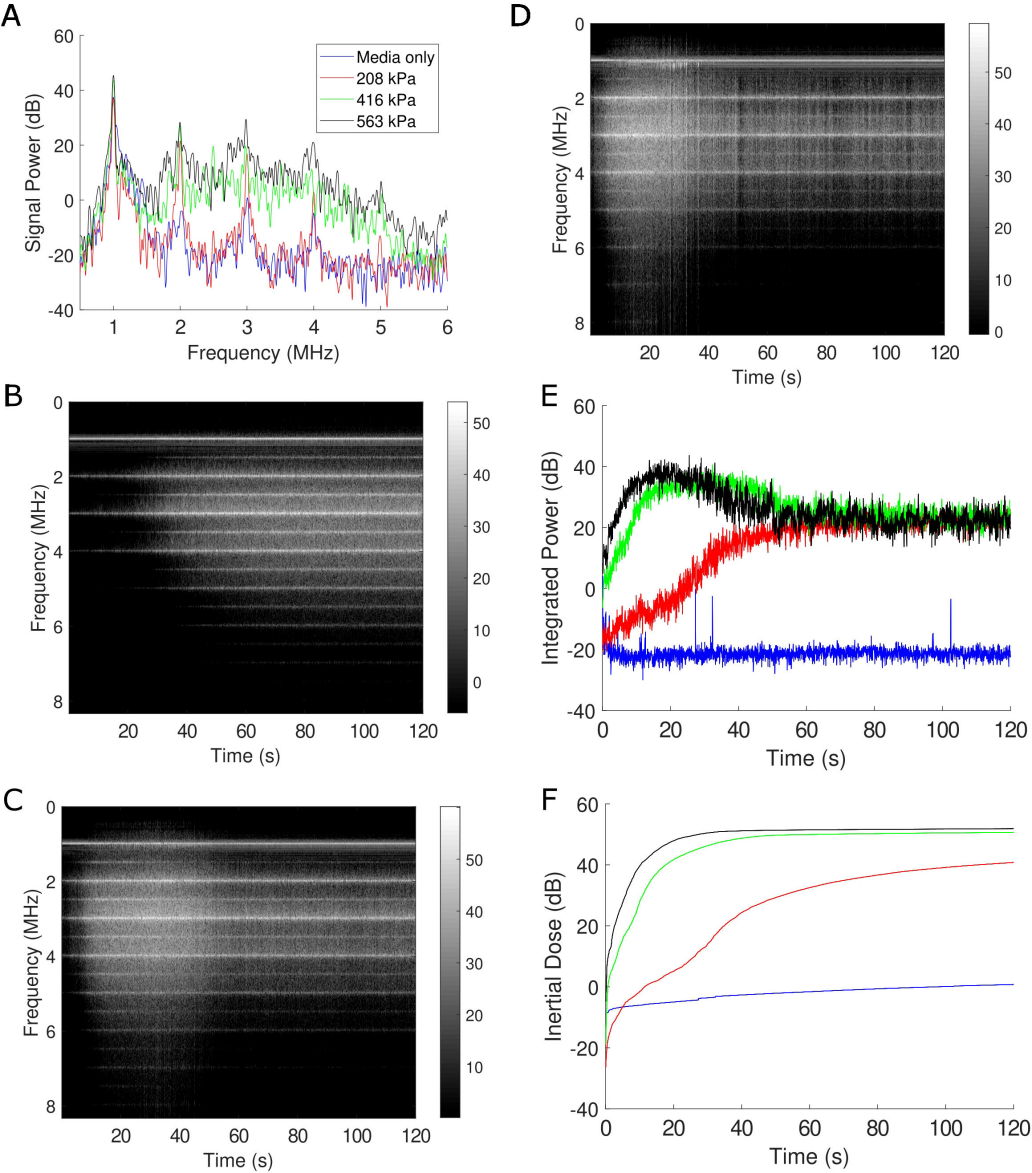
Passive cavitation data confirm microbubble disruption during treatment. **(A)** A representative sample of the Fourier spectrum of the received echo data for the three acoustic conditions employed here, as well as a sham treatment all at *f*=1 MHz. Clear harmonic (*nf* with *n*=1,2,3…) and broadband emissions (in-between the harmonic components) under treatment conditions are evident at this time-point (1 s into treatment). The level of broadband emission is a well-known surrogate metric for microbubble disruption. **(B-D)** Joint time-frequency analysis of the 208 kPa, 416 kPa, and 563 kPa treatment, respectively. In these panels, the FFT data is represented on the vertical axis over treatment duration (120 s), with the grayscale encoding the magnitude of signal power (in dB). Spectral lines corresponding to harmonic and subharmonic (*nf*/2 with *n*=1,3,5…) energy are visible, with increasing propensity of broadband emissions at increasing pressures. **(E)** The integrated power corresponding to broadband emissions over time and **(F)** its corresponding cumulative dose. See text for details.

To appreciate the corresponding and correlative bubble physics, the microbubble echo was simultaneously recorded during treatment using passive cavitation detection, as depicted in **Figure 4**. A representative frequency spectrum, which illustrates the frequency content of the microbubble signal at a given point in time (*i.e. t*=1s), is shown in **Figure 4A** for the three acoustic pressures employed along with a cell culture media only control. Here, the media-only control (*i.e.* with no microbubbles, shown in blue) is characterized by reflections picked up by the passive transducer mainly at the transmit frequency (*f*=1 MHz) and arising from the boundaries of the acoustic chamber. With increasing acoustic pressure, the presence of harmonic signal (*nf* with *n*=2,3,4…) emerges, along with broadband emissions characteristic of microbubble collapse. Stable harmonic signal – including at ultra-harmonic frequencies (*nf/2* with *n*=3,5,7…) - is consistent throughout the two-minute treatment time, illustrated via the time-frequency analysis plots at 208 kPa (**Figure 4B**), 416 kPa (**Figure 4C**) and 563 kPa (**Figure 4D**). Most notably at the higher acoustic pressures, broadband emissions are detectable in these representations by a smearing of signal across the spectrum and represents progressive microbubble disruption. The integrated power of this broadband emission is quantified in **Figure 4E**, which highlights bubble collapse at 208 kPa (red trace) and more abrupt and sustained collapse for 416 kPa (green trace) and 563 kPa (black trace). As an overall quantification of microbubble collapse throughout the treatment, the inertial cavitation dose (24, 25, 36) (cumulative integrated power; **Figure 4F**) confirms the time evolution of microbubble disruption through the treatment time.

**Figure 5:**
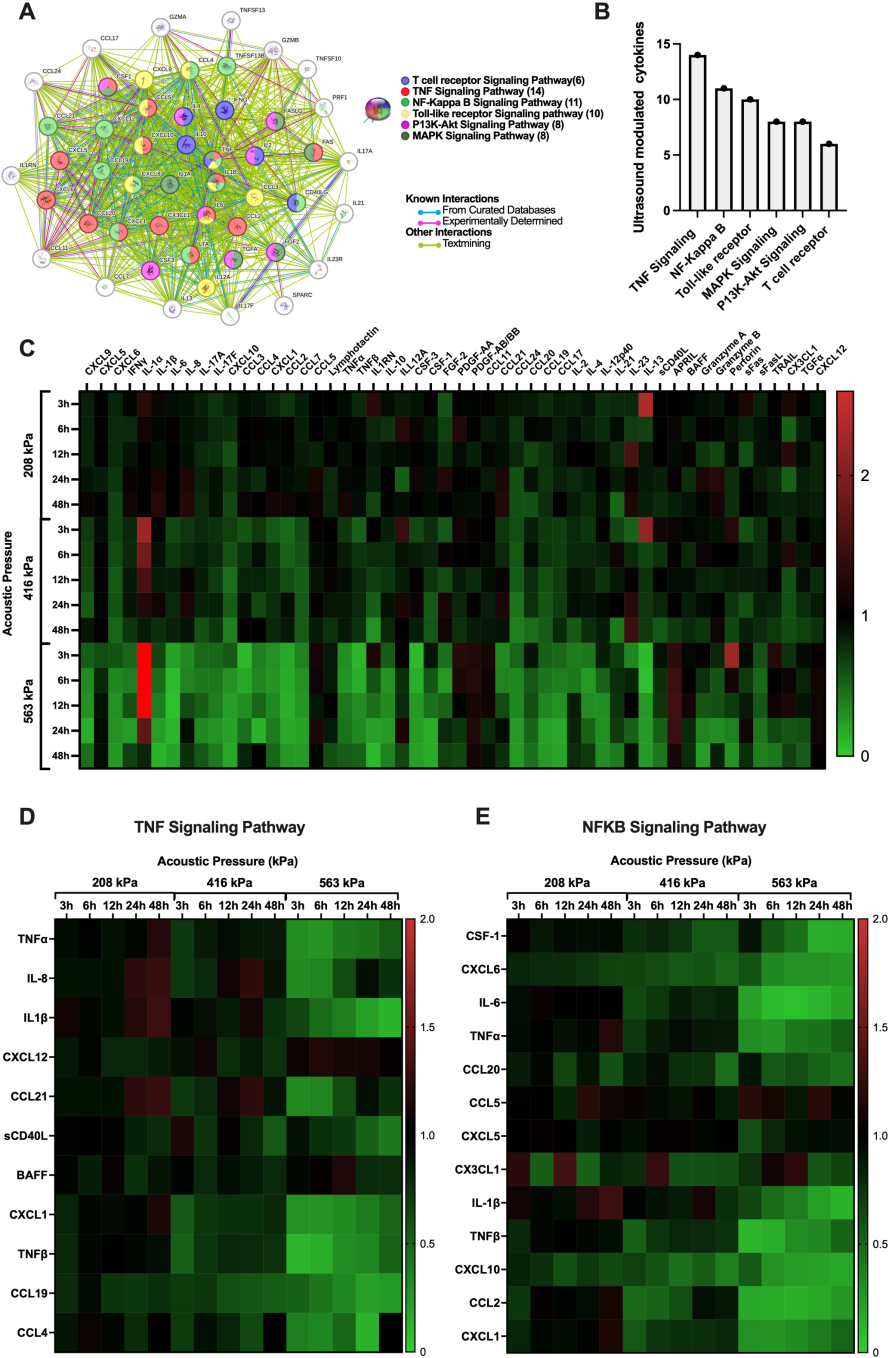
Microbubble-mediated treatment of immune cells elicits time-dependent changes to cytokine and other analyte secretion that are highly dependent on the transmit acoustic pressure. **(A)** String protein interaction map showing the functional links and pathways between analytes that were affected by ultrasound treatment as compartmentalized using the KEGG database collection, globally summarized via the **(B)** number of proteins that were modulated in a few major pathways related to cancer. Note that the TNF and NFκB pathways are the most affected by ultrasound, in terms of the absolute number of relevant analytes. All pathway connections here are characterized by false discovery rates *p*<0.001. See text for details. **(C)** Heatmap with all significantly modulated cytokine fold changes in PBMCs. **(D)** Heatmaps within the TNF and **(E)** the NFκB signaling pathway depict the effect and immunomodulation of microbubble-mediated focused ultrasound treatment on the secretome of PBMCs. Fold increase in cytokine production compared to sham controls is depicted as red and a decrease is depicted as green. Significance is determined as *p*<0.05.

With the acoustic conditions and bubble physics that elicit very minor (208 kPa), moderate (416 kPa) and significant (563 kPa) immune cell plasma membrane perforation in hand, we next sought to look beyond the membrane to examine the effects of these acoustic conditions on the immune cell secretome in a time-dependent manner. Indeed, the microbubble-mediated treatment of PBMCs demonstrated significant, time-dependent changes in cytokine and analyte secretion which were highly dependent on acoustic pressure. We synthesized a protein interaction map (**Figure 5A**) to illustrate the complex functional links and pathways between analytes that were significantly affected by ultrasound treatment at any pressure and any time point. Here, the circles represent a given cytokine and the colored lines between them represent known interactions either from curated databases (blue), experimental determination (purple) or text mining (green). Curated databases provide high-confidence interactions verified by expert review, experimental determination indicates interactions observed through laboratory experiments, and text mining involves identifying interactions from published scientific literature using computational methods (37). The color of the circle itself gives an indication as to which common pathway the cytokine modulates or is modulated by, including functional pathways in T-cell receptor signaling, TNF signaling, NF-κB signaling, Toll-like receptor (TLR) signaling, PI3K-Akt signaling, and MAPK signaling. The global summary (**Figure 5B**) depicts the number of proteins modulated in these major pathways, with TNF signaling exhibiting the highest modulation (14 cytokines), followed by NF-κB (11 cytokines), TLR (10), PI3K-Akt (8), MAPK (8) and T-cell receptor (8) signaling pathways. To illustrate the entirety of ultrasound-modulated PMBC secretome modulation, **Figure 5C** shows a heatmap presenting the fold changes in cytokine production over various time points post-ultrasound (3h, 6h, 12h, 24h, and 48h) and acoustic pressures (208 kPa, 416 kPa, and 563 kPa). Here, an increase in cytokine production at any given time point compared to sham controls at that time point is depicted in red, while a decrease is shown in green. Only the cytokines that exhibited a statistically significant fold-change for at least a single time point at *p* < 0.05 are included in this figure. As can be seen from this heatmap, microbubble treatment exerted a rather heterogeneous response across the secretome spectrum - ranging from 0.06-fold decrease (IL-13 at 6 hours) to a 3.8-fold increase (IL-1α at 3 hours) post ultrasound treatment at 563kPa. At 208 kPa, for example, there was an increase in the production of several cytokines at early time points (3h and 6h) such as IL-1α and IL-13, but these changes were not as pronounced as those observed at higher acoustic pressures. As the acoustic pressure increased to 416 kPa and 563 kPa, a more substantial and widespread modulation of cytokine secretion was evident. Specifically, at 563 kPa, there was a significant increase in fold change of some cytokines such as IL-1α and perforin at 3h post ultrasound (3.8 and 2.2-fold respectively) as well as a significant decrease in certain cytokines such as IL-6 at 12h post-ultrasound or TNF-α at 6h post-ultrasound (0.07 and 0.3-fold respectively). The highest pressure elicited both downregulation and upregulation of different cytokines, with more evident downregulation occurring at later time-points. The heatmap reflects these global observations, confirming that higher acoustic pressures result in more pronounced changes in cytokine production.

To drill a little deeper into the data, we highlight in **Figures 5D, E** the differential expression of cytokines involved in the two most prevalent/modified pathways (shown in **Figure 5B**) - the TNF and NFκB signaling pathways – across all acoustic pressures (208 kPa, 416 kPa, 563 kPa) and time points (3h, 6h, 12h, 24h, 48h) employed here. The TNF and NFκB pathways are crucial in cancer and T-cell function. Indeed, TNF signaling is vital for T-cell activation and differentiation, influencing the immune system’s ability to target and destroy cancer cells (38)​; while the NFκB pathway is a central mediator of immune response and inflammation and plays a significant role in cancer progression by regulating genes involved in cell survival, proliferation, and metastasis​ (39). Specifically, we explore the temporal evolution of a subset of analytes involved in key signaling activities, from inflammation to T cell chemotaxis and activation – fully acknowledging the complexity of this landscape: from the potential pleiotropic effects of these analytes on immune cells, endothelial cells, and cancer cells alike, to the differential response of these cytokines within the context of different types of cancers and tumor microenvironments.

**Figure 6:**
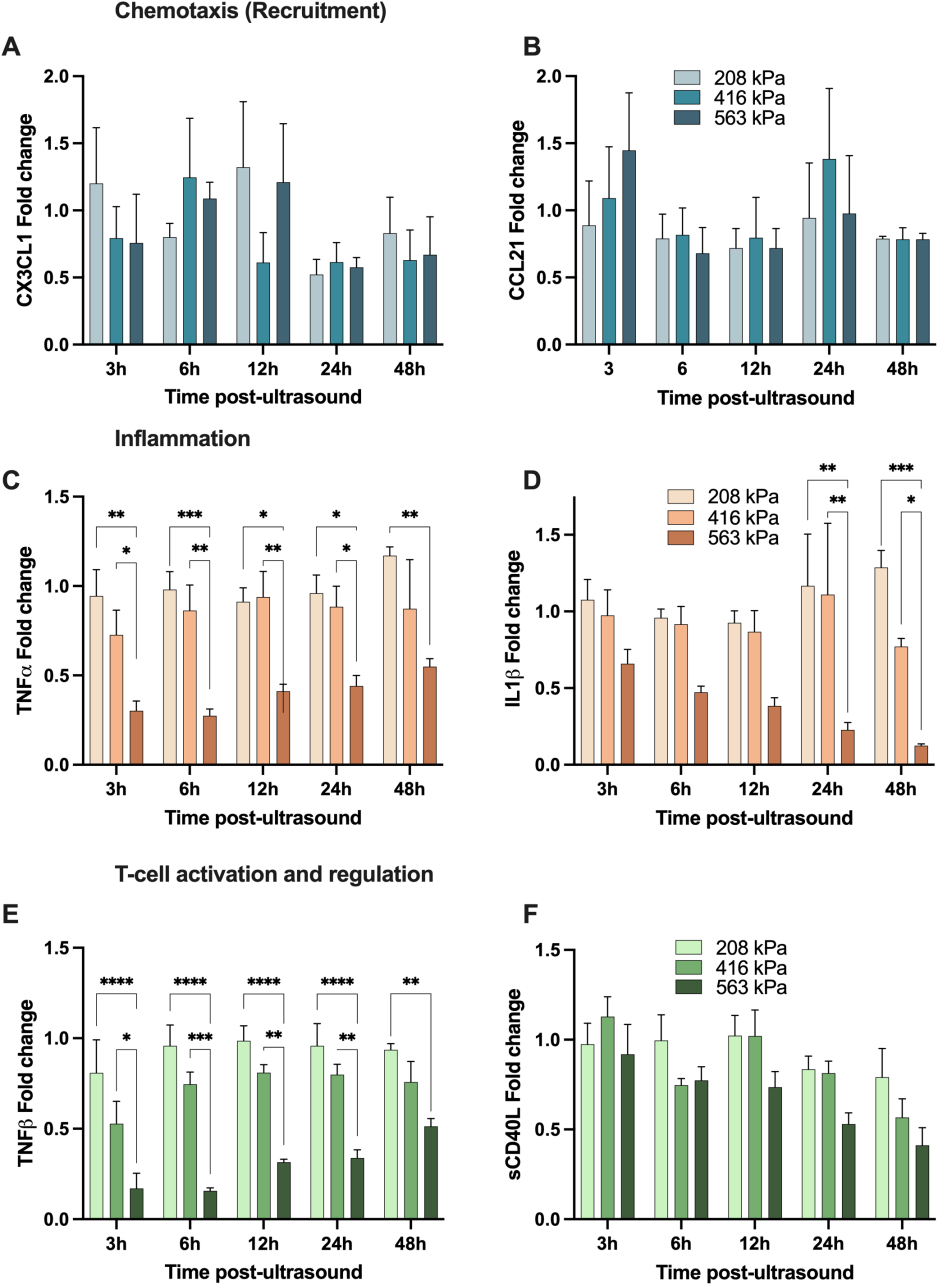
Focused ultrasound can modulate the secretion of analytes with chemotactic, inflammatory and T-cell regulation function. All panels depict the fold-change of a given cytokine with respective to its sham time-matched control. The top two panels demonstrate examples of cytokines involved in immune cell chemotaxis; **(A)** CX3CL1 and **(B)** CCL21; while the middle panels show samples of cytokines involved in inflammation; **(C)** TNFα and **(D)** IL-1β, and finally the bottom two depict analytes with T-cell activation and regulation function; **(E)** TNF-β (LTα) and **(F)** soluble CD40L. Note that these secretions are both time-dependent and acoustic pressure-dependent, and these are secreted from the freshly isolated human peripheral blood mononuclear cells. Asterisks here denotes significance via a two-way ANOVA * *p*<0.05, ** *p*<0.01, *** *p*<0.001, **** *p*<0.0001. See text for details.

We first begin with the focused ultrasound modulation of fractalkine (CX3CL1; **Figure 6A**) which was demonstrated to be heterogeneous, with an increase to in its secretion relative to the sham group at earlier time points (<12 hours), followed by a decrease towards 48 hours post-treatment, regardless of acoustic pressure used. Indeed, there are conflicting studies on the role of CX3CL1 with regards to anti-tumor immunity; with some investigations concluding that it is a potent recruiter of NK and T cells into the TME (40, 41), and others showing increased recruitment of myeloid-derived suppressor cells (MDSCs) and identifying positive correlations between increased intertumoral CX3CL1 concentrations and tumor severity (42, 43). Next CCL21, a multifaceted role in the tumor microenvironment (TME), influencing immune responses and cancer progression through various mechanisms. It has been shown to affect the polarization of neutrophils, crucial for determining the immunotherapy response in cancers such as hepatocellular carcinoma (44). It promotes activity in the TME through the co-localization of dendritic cells and T-cells (45). High levels of CCL21 correlate with increased infiltration of immune cells like neutrophils, CD8+ T-cells, and macrophages, indicating a more reactive immune environment in tumors (45, 46). This suggests that tumors with higher CCL21 expression might be more susceptible to immunotherapeutic strategies and show that higher levels of CCL21 also improve outcomes of T-cell adoptive cell transfer therapies (47). Here, we show an increase of CCL21 3h post-ultrasound at the highest pressure 563kPa (1.4-fold) while at the intermediate pressure that increase occurs 24h post-ultrasound - although it eventually returns to baseline sham levels (**Figure 6B**).

TNF-α – which is involved in inflammation and host defense – can exhibit paradoxical dual roles in the progression and treatment of cancer; with high local doses result in tumor vasculature destruction, and more steady, low-level and chronic secretion acting as an endogenous tumor promoter (48–50). Similar to IL-1β, our focused ultrasound treatment at the highest acoustic pressure resulted in a decrease in TNF-α secretion (0.3-0.5-fold from sham levels from 3-48 hours), although exhibiting a transient response as TNF-α concentration increased closer to baseline over time (**Figure 6C**). IL-1β is a potent and quintessential pro-inflammatory cytokine with documented roles in cardiovascular diseases (51) and cancer (52). Within the context of the TME, numerous pre-clinical cancer models have demonstrated that high local concentrations of IL-1β contribute towards immunosuppression by stimulating the recruitment of MDSCs (53, 54), modifying regulatory T cell response (55), and promoting angiogenesis and tumor metastasis (56). Further, elevated IL-1β levels in patient tumors (along with IL-6 and IL-8, which in turn are regulated by IL-1β) have been detected over a wide range of cancers (57). Here, we highlight IL-1β secretion progressively decreases post ultrasound at the highest acoustic pressure employed, while approximately maintaining its native sham levels at the two lowest acoustic conditions (**Figure 6D**), with IL-6 following a similar pattern (see **Figure 5E**).

Lymphotoxin-alpha (LTα), also known as TNF-β, has complex roles in the tumor microenvironment, similarly to TNF-α. It shares receptors with TNF-α, influencing inflammation and immune cell function through both TNFR1 and TNFR2. Its interactions with these receptors can either promote or inhibit tumor progression, depending on the context within the tumor microenvironment (58). After ultrasound treatment, our data suggests that TNF-β experiences a transient down-regulation in higher and intermediate pressures and then returns to sham baseline levels over time (**Figure 6E**). This down-regulation is larger for the highest pressure of 563kPa (0.2-fold 3h post-ultrasound) compared to 416kPa where the cytokine levels go down 0.5-fold 3h post-ultrasound. After 48h TNF-β levels raised to 0.5-fold (563kPa) and 0.8-fold (416kPa), which is slightly lower than sham baseline. The transient suppression might temporarily reduce the pro-inflammatory signals within the TME, potentially decreasing the recruitment and activation of immune cells that could otherwise support tumor growth. However, the reduction in TNF-β could also temporarily impede the formation of new blood vessels (angiogenesis), a crucial process for tumor growth and metastasis. Understanding and manipulating the kinetics of TNF-β secretion after therapeutic interventions like ultrasound could be beneficial for designing treatment protocols that optimize anti-tumor immunity and minimize tumor-promoting inflammation.

Soluble CD40 ligand (sCD40L) plays a significant role in the tumor microenvironment (TME), particularly in modulating immune responses and influencing tumor progression. sCD40L, primarily expressed by activated CD4+ T cells, binds to the CD40 receptor on antigen-presenting cells (APCs). This interaction is crucial for T-cell priming and enhances the immune system’s ability to combat tumors by promoting cytokine production and strengthening the innate immune response. Moreover, studies have shown that sCD40L can directly suppress tumor cell proliferation and induce apoptosis in certain cancer cells. This suggests a potential therapeutic role for sCD40L in inhibiting tumor growth directly​ (59). The interaction between CD40L and CD40 also has implications for TGF-β production, which can enhance the immunosuppressive function of cancer cells, contributing to tumor progression. Thus, while CD40L has potential therapeutic benefits, its role in cancer is complex, and its effects can vary depending on the context within the TME (60). Here, we observe that focused ultrasound decreases sCD40L over time at all acoustic pressures employed here; for example down to 0.4-fold 48h post ultrasound at the highest pressure condition (**Figure 6F**).

**Figure 7:**
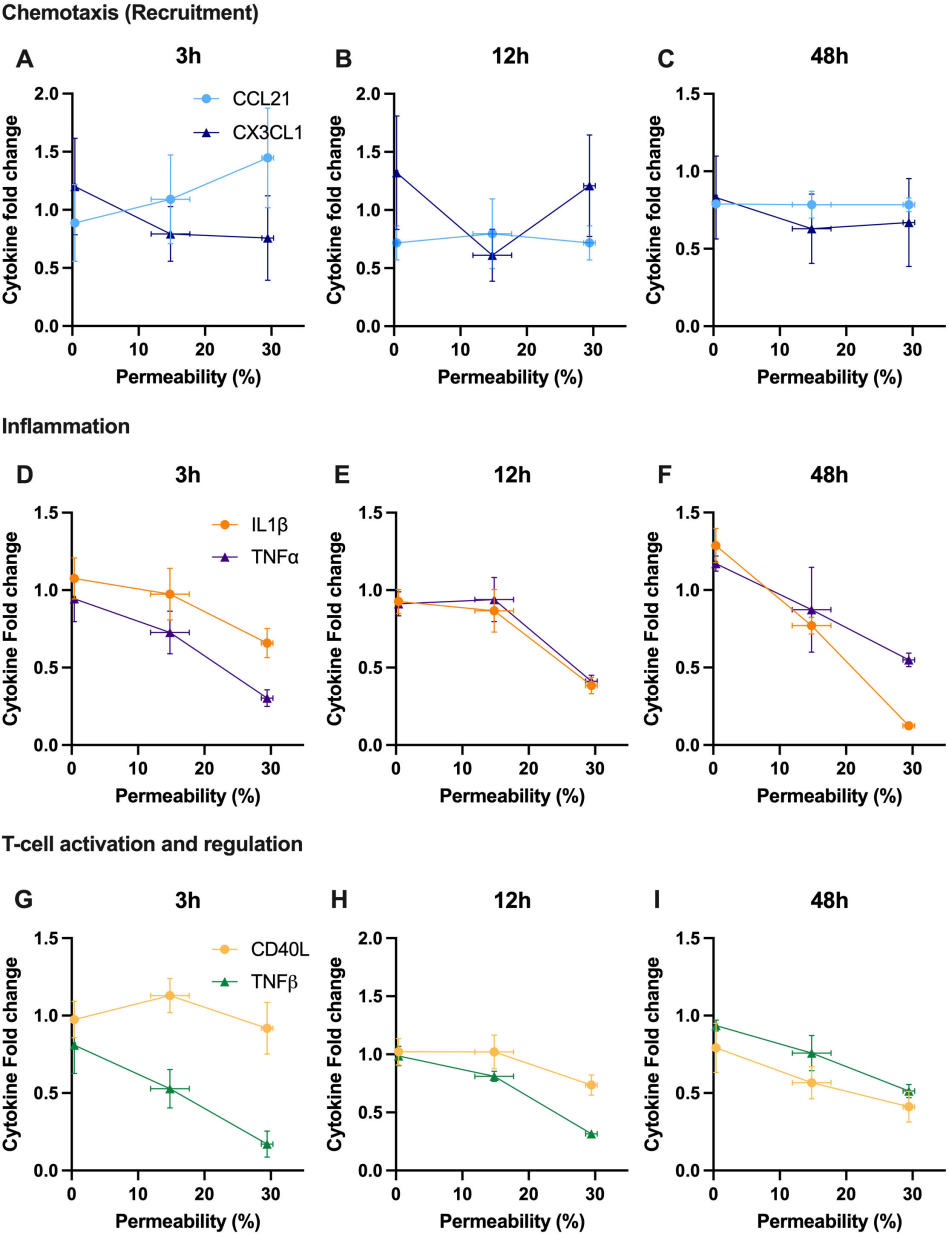
Secreted cytokine content is correlated with acoustically induced plasma membrane permeability, mostly exhibiting a negatively correlated relationship at a given time post therapy. A snapshot ranging from 3 to 48 hours post-ultrasound of relative cytokine secretion as a function of enhanced permeability of PBMCs (as measured by FITC-dextran uptake). Note that the selected cytokines are the same as depicted in **Figure 6**, and the fold-change is with respect to the sham treatment. Cytokines involved in chemotaxis are shown at **(A)** 3 hours, **(B)** 12 hours, and **(C)** 48 hours, including CCL21 and CX3CL1. Cytokines involved in inflammation are depicted at **(D)** 3 hours, **(E)** 12 hours, and **(F)** 48 hours, with TNF-α and IL-1β. Cytokines involved in T-cell activation and regulation are presented at **(G)** 3 hours, **(H)** 12 hours, and **(I)** 48 hours, highlighting TNF-β and soluble CD40L. R-value was calculated using Pearson’s correlation coefficient.

Finally, we aim to correlate the modulation of key cytokines involved, in a broad sense, in immunomodulation with acoustically induced immune cell membrane permeability (**Figure 7**). Here, each of the cytokines presented in **Figure 6** are shown at either 3h, 12h or 48h as a function of our PMBC population study on enhanced plasma membrane permeability (**Figure 3**). From this dataset, it can be seen that, with a few exceptions, the predominate relation is one of negative correlation; that is to say that increasing levels of reversible plasma membrane perforation tends to result in decreasing cytokine production at a given time-point post-focused ultrasound therapy. Linear regression analysis highlights a few statistically significant negative correlations, as summarized in **Table 1**, with correlation coefficients ranging from 0 ≤ |*R*| ≤ 0.954.

**Table 1:** A subset of cytokine expression profiles statistically correlate with focused ultrasound induced membrane permeability.

Cytokine	*R* value			*p* value		
	3h	12h	48h	3h	12h	48h
CCL21	0.151	<0.01	<0.01	0.30	0.99	0.94
CX3CL1	0.116	<0.01	0.03	0.37	0.86	0.65
IL-1β	0.492	0.634	0.954	0.05	0.01	<0.0001
TNF-α	0.688	0.543	0.549	<0.01	0.02	<0.02
sCD40L	0.01	0.302	0.437	0.77	0.12	0.05
TNF-B	0.645	0.866	0.733	<0.01	<0.001	<0.01

Listed here at the *p* values of the linear regression analysis performed on the data from **Figure 7**. Entries in red signify *p*<0.05. Note that the correlation coefficients ranged from 0 ≤ |*R*| ≤ 0.954. Linear regression was analyzed using Pearson’s correlation coefficient.

It is important here to note that we acknowledge several study limitations. Firstly, the use of freshly isolated PBMCs renders a detailed analysis of immune-cell specific (*e.g.* T cell subtype) cytokine release challenging. Further, as previously mentioned, the interpretation of the roles of each analyte and its overall function is context-dependent (*e.g.* presence of other cell types, or other circulating factors), with many cytokines/chemokines exhibiting paradoxical roles in anti-tumor immunity. Indeed, several cytokines secreted by cells within the TME exhibit potent anti-immune responses that may confound the overall effectiveness of the ultrasound-mediated cytokine release demonstrated here, and further *in-vivo* investigations are necessary to robustly characterize this effect. We also acknowledge the conceptual limitation here that there exist many focused-ultrasound conditions in which, despite exerting no measurable membrane perforation, alter intracellular calcium influx and modulate downstream NF-κB signaling (13, 61). Indeed, this avenue of membrane perforation-independent secretome modulation warrants further investigation.

## 4 Conclusions

We have demonstrated here that microbubble-assisted focused ultrasound modulates immune cells both in terms of enhanced cell membrane permeability and the secretion of pro-immune cytokine and chemokines. Indeed, secretome analysis revealed time-dependent alterations in cytokine and chemokine profiles, implicating key signaling pathways in immune response modulation, such as NFκB and TNF pathways, and modifying the concentrations of important analytes including IL-1β, TNF-α, TNF-β, CCL21, CX3CL1, and soluble CD40L – among others. Taken together, this data suggests that microbubble-mediated focused ultrasound modulation of human immune cells can change local concentrations of key secretions that may improve the efficacy of cancer immunotherapy.

## Data Availability

The raw data supporting the conclusions of this article will be made available by the authors, without undue reservation.
