# Supplementary material for: Immunomodulation of human T cells by microbubble-mediated focused ultrasound
Source: Front Immunol. 2024 Oct 22;15:1486744. doi: 10.3389/fimmu.2024.1486744 (PMC11534865; doi:10.3389/fimmu.2024.1486744)
Supplement: Supplementary file 1 [file DataSheet1.docx]

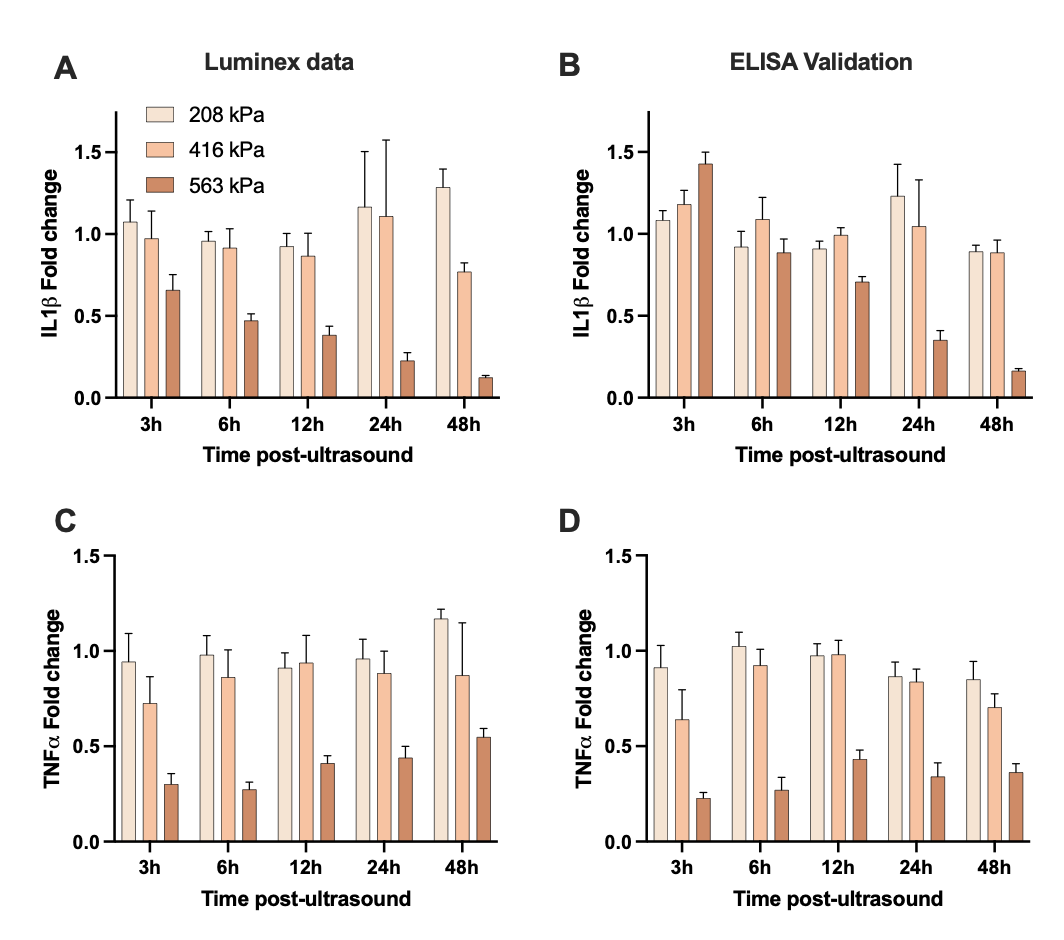


**Figure S1.** The effect of ultrasound on the immune cell release of A,B) IL-1β and C,D) TNFα, as measured by the Luminex multiplex assay (left – reported in the manuscript) and validated by individual ELISA assays (right). The ELISA data confirms the overall trends provided by the multiplex data.
